# Altered functional connectivity and topology structures in default mode network induced by inflammatory exposure in aged rat: A resting-state functional magnetic resonance imaging study

**DOI:** 10.3389/fnagi.2022.1013478

**Published:** 2022-11-17

**Authors:** Yang Liu, Huiru Feng, Huiqun Fu, Yan Wu, Binbin Nie, Tianlong Wang

**Affiliations:** ^1^Department of Anesthesiology, Xuanwu Hospital, Capital Medical University, Beijing, China; ^2^National Clinical Research Center for Geriatric Diseases, Beijing, China; ^3^Department of Anatomy, Capital Medical University, Beijing, China; ^4^Institute of High Energy Physics, Chinese Academy of Sciences, Beijing, China

**Keywords:** anesthesia, inflammation, cognitive dysfunction, default mode network, small worldness

## Abstract

Inflammatory stress in anesthesia management and surgical process has been reported to induce long-term cognitive dysfunction in vulnerable aged brain, while few studies focused on the network mechanism. The default mode network (DMN) plays a significant role in spontaneous cognitive function. Changes in topology structure and functional connectivity (FC) of DMN in vulnerable aged brain following inflammatory stress-induced long-term cognitive dysfunction are rarely studied. Eighty-eight aged male rats received intraperitoneal injection of lipopolysaccharide (LPS) as treatment or equal amount of normal saline (NS) as control. Morris Water Maze (MWM) was performed to assess short- (<7 days) and long-term (>30 days) learning and spatial working memory. Enzyme-linked immunosorbent assay (ELISA) was used to measure systemic and hippocampus inflammatory cytokines. Real-time polymerase chain reaction (RT-PCR) was used to measure the changes in gene level. Resting-state functional magnetic resonance imaging (rs-fMRI) was used to exam brain function *prior* to MWM on days 3, 7, and 31 after LPS exposure. Graph theory analysis was used to analyze FC and topology structures in aged rat DMN. Aged rats treated with LPS showed short- and long-term impairment in learning and spatial working memory in MWM test. Graph theory analysis showed temporary DMN intrinsic connectivity increased on day 3 followed with subsequent DMN intrinsic connectivity significantly altered on day 7 and day 31 in LPS-exposed rats as compared with controls. Short- and long-term alterations were observed in FC, while alterations in topology structures were only observed on day 3. Rats with inflammatory stress exposure may cause short- and long-term alterations in intrinsic connectivity in aged rat’s DMN while the changes in topology structures only lasted for 3 days. Inflammatory stress has prolonged effects on FC, but not topology structures in venerable aged brain.

## Introduction

Cognitive dysfunction following anesthesia and surgery is widely reported in clinical practice (Belrose and Noppens, 2019; Liu et al., 2022). Among the population, elderly patients were shown to be more vulnerable to neuroinflammation induced by anesthesia neurotoxicity and surgical trauma, which may further develop into long-term cognitive dysfunction (Daiello et al., 2019). Surgical trauma is recently believed to be a major source for inflammatory cytokines in clinical practice (Yang T. et al., 2020; Liu et al., 2022). Clinical study has shown that the postoperative cognitive decline is associated with increased mortality (Yang T. et al., 2020; Duprey et al., 2021; Liu et al., 2022). In this case, understanding how the cognitive impaired aged brain functions in such conditions is of great significance for disease prevention and drug target design, as well as a better perioperative outcome for elderly patients.

Traditional neuroscience approaches mainly focused on several localized regions (e.g., hippocampus and prefrontal cortex; Lisman et al., 2017; Le Merre et al., 2021) to make a detailed experiment in particular animal models. However, as none of the cognitive function can be fully understood if taken out of a broad connectionist context (Fuster, 2001) and the brain functions as networks instead of separate regions in living subjects (Avena-Koenigsberger et al., 2017), results from traditional approaches may only provide limited evidence in disease mechanism and development. Recent studies reported the significance of “neural network disease,” as the functions of neural network and topology changes contributed largely to disease development and revealed medical treatment outcomes (Zhang et al., 2021; Kukla et al., 2022). These results highly emphasized the importance of understanding how the neural network changes during inflammatory exposure induced cognitive decline in a broad scale in living brain.

Resting-state functional magnetic resonance imaging (rs-fMRI), which measures spontaneous brain activity *via* low frequency fluctuations in blood-oxygen-level-dependent (BOLD) signal, provides a non-invasive method to study brain function in living subjects (Biswal et al., 1995). By abstracting brain networks as graphs, the graph theory method analyzed BOLD signal by dividing the brain networks into different elements (nodes) and their pairwise links (edges). Brain nodes are neurons/entire brain regions and edges are binary/weighted values (Sporns, 2018). Functional connectivity (FC), defined as correlations (edges in the graph) of neuronal activation between different brain regions (nodes in the graph), has been shown to be modulated in various diseases conditions (Ji et al., 2018; Liu et al., 2018; Schumacher et al., 2021). Meanwhile, topology properties and intrinsic connectivity of brain networks may also be altered in disease situations (Lopes et al., 2017; Lee et al., 2019). In this way, by considering topology properties and FC in a whole scale, rs-fMRI and graph theory method provide a detailed understanding of changes in brain networks, indicating an optional choice for studying disease mechanisms.

The default mode network (DMN) is a set of widely distributed brain regions, which often show reductions in activity during attention-demanding tasks but increase their activity across multiple forms of complex cognition. Many of which are linked to memory or abstract thought (Smallwood et al., 2021). Impaired FC in DMN has been characterized as one of the major changes in patients with Alzheimer’s disease (AD) and mild cognitive impairment (MCI; Chand et al., 2017; Hohenfeld et al., 2018; Cao et al., 2020), demonstrating a potential use for FC in DMN in cognitive function. Recent data from preclinical experiments have shown that the DMN not only exists across species but also similar in components (Li and Zhang, 2018; Jing et al., 2021; Yeshurun et al., 2021), suggesting that study of DMN in rodent models would be applicable in investigating mechanisms of human diseases. Although there are studies demonstrating changes in DMN in Aβ-induced cognitive decline (Pascoal et al., 2019), few studies focused on the DMN alteration in acute- and long-term after inflammatory stress exposure.

This study is designed to investigate acute and long-term changes in DMN after inflammatory stress exposure in anesthesia process. Inflammatory stress was induced by lipopolysaccharide (LPS) injection, as it is one of the most widely used initiators for inducing systemic inflammatory responses and cognitive decline (Kan et al., 2016; Lakshmikanth et al., 2016; Yang L. et al., 2020) in preclinical experiments. Aged rats were used in this experiment because elderly patients are at highly risk for cognitive dysfunction after anesthesia and surgery (Belrose and Noppens, 2019). The model was adapted from our previous work, which demonstrated that a single intraperitoneal injection of LPS (2 mg/kg) induces a long-term inflammatory response in aged rat hippocampus and impaired behavior performances (Fu et al., 2014; Kan et al., 2016). Systemic levels of inflammatory cytokines, including interleukin-1β (IL-1β) and tumor necrosis factor-alpha (TNFα), were measured by enzyme-linked immunosorbent assay (ELISA) as their levels are highly correlated with sickness behavior after LPS exposure (Oliveira et al., 2020). We hypothesized that the topology structure and FC in the aged rat DMN will both change after inflammatory exposure, which may provide new insights into drug target design and new diagnostic biomarkers.

## Materials and methods

### Ethics approval

All animal procedures were approved by the Ethical Committee of Capital Medical University as complied with the guide for Care and Use of Laboratory Animals prepared by the Institute of Laboratory Animal Resources and published by the National Institute of Health. All efforts were made to minimize the pain and suffering of the animals.

### Animal preparations

A total of 88 adult male Wistar rats (19 months, 650–800 g, Charles-River Animal Technology, Beijing, China) were housed in an animal facility under a 12/12 light–dark cycle at 22 ± 2°C with 50%–60% humidity. Food and water were available *ad libitum*.

**Figure 1:**
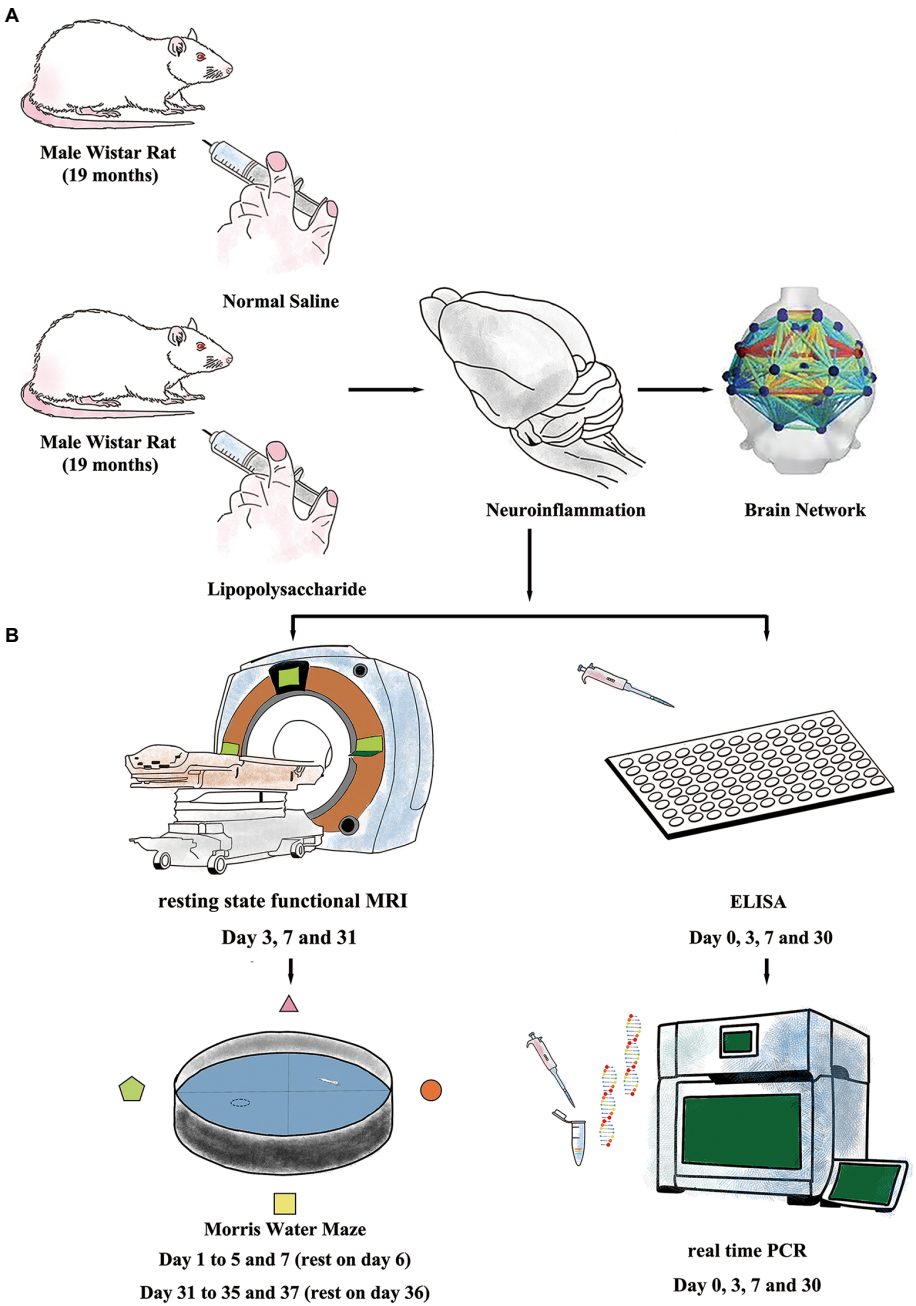
Experiment protocols. **(A)** Inflammatory exposure was induced by intraperitoneal injection of lipopolysaccharide (LPS, 2 mg/kg) in aged rat. The LPS further induced short- and long-term neuroinflammation and resulted in impaired brain network. **(B)** Resting-state-functional magnetic resonance imaging (rs-fMRI) and Morris Water Maze (MWM) were used to evaluate changes in neural network and cognitive function. Enzyme-linked immunosorbent assay (ELISA) and real-time polymerase chain reaction (RT-PCR) were performed to study neuroinflammation.

Rats were intraperitoneally injected (i.p.) with either lipopolysaccharide (LPS, 055: B5, Sigma-Aldrich, St Louis, MO, United States) or sterilized normal saline (NS, OC90F1, Otsuka Pharmaceutical, Co, Ltd., Beijing, China) and were allocated to either LPS group (*n* = 60, 2 mg/kg, i.p.) or NS group (*n* = 28, same volume with LPS solution injection). The dose for LPS was adapted from our previous work which showed that low-dose LPS injection caused prolonged cognitive dysfunction in aged rat (Figure 1A; Fu et al., 2014; Kan et al., 2016).

### Morris water maze

**Figure 2:**
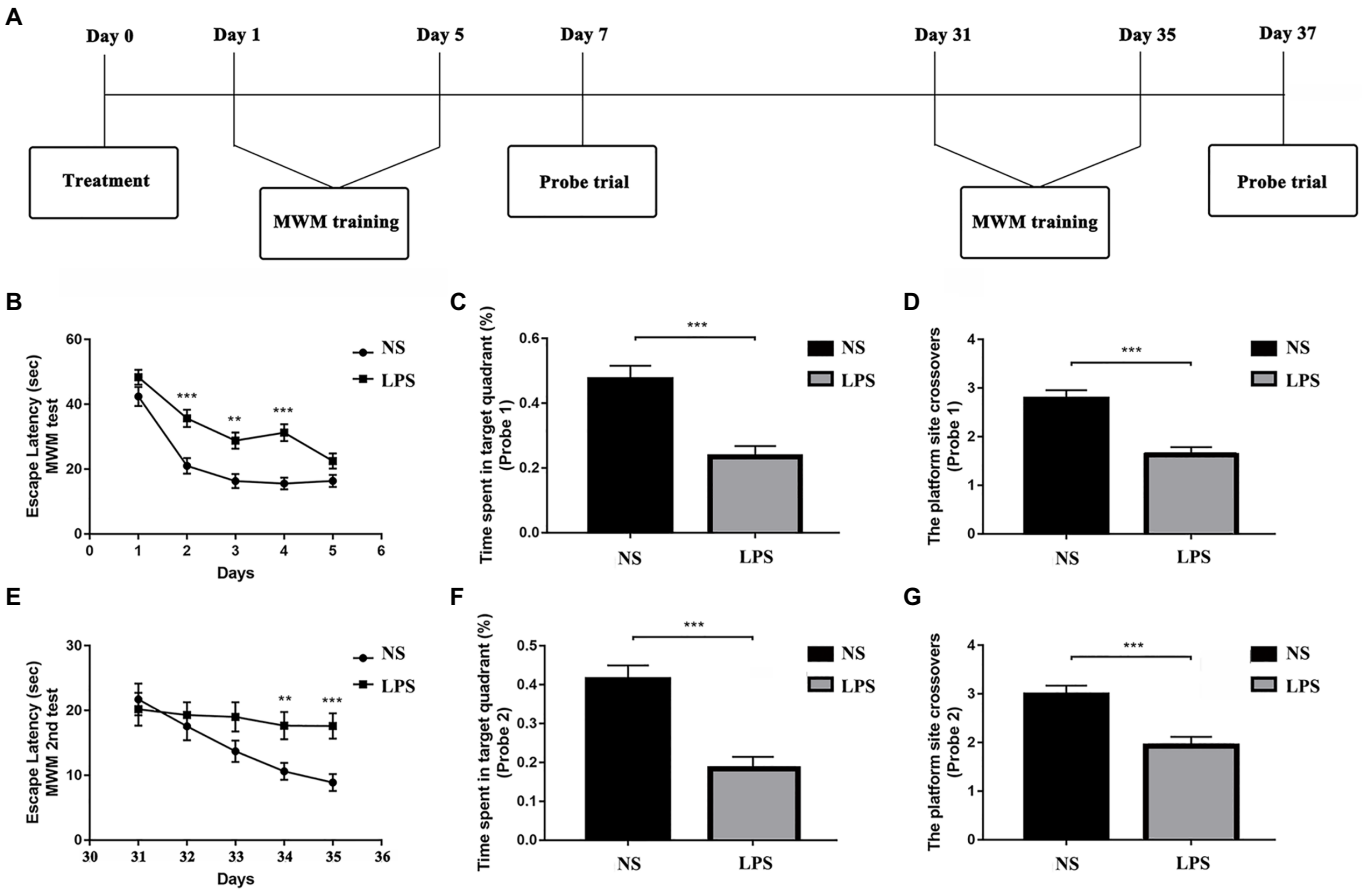
Lipopolysaccharide (LPS) exposure caused short- and long-term impaired learning and spatial working memory. **(A)** Morris Water Maze (MWM) test protocol. **(B)** Rats with LPS exposure had longer escape latency compared with those in the control group from day 2 to day 4. **(C,D)** Rats with LPS exposure spent shorter time in the target quadrant and performed fewer platform site crossovers compared with those in the control group. **(E)** Rats with LPS exposure had longer escape latency compared with those in the control group from day 34 to day 35. **(F,G)** Rats with LPS exposure spent shorter time in target quadrant and performed fewer platform site crossovers compared with those in the control group. Escape latency was analyzed by two-way ANOVA for repeated measurement followed by Bonferroni *post hoc* analysis. Time spent in the target quadrant and number of crossovers were analyzed using two-sample *t*-test. Data were presented as mean ± standard error of mean (SEM). ^*^p < 0.05, ^**^p < 0.01, ^***^p < 0.001 vs. control group.

All rats (*n* = 12 in each group) performed MWM test with the protocols adopted from our previous work (Kan et al., 2016). The MWM is a circular tank placed in a quiet and dimly lit room with visual clues around. The inner surface of the tank was painted in black and filled with water (24 ± 1°C) in about 20 cm in depth. A hidden platform was placed in one fixed quadrant hidden in about 1.5 cm below the water surface. On top of the circular tank is a video camera tracking the behavior performance of the rats. Around the circular tank is a series of large support holders with thick curtains to avoid sharp lights if necessary. Briefly, the MWM test contains two sections of spatial acquisition trials (day 1–5 and day 31–35) and probe trials (day 7 and day 37) to assess learning and spatial working memory in both short term (<7 days) and long term (>30 days). For the spatial acquisition trial, the rat was gently placed in one of the quadrants facing the wall of MWM circular pool and then was allowed to swim freely for 60 s to find the hidden platform. If the rat succeeds, it was allowed to stay on the platform for 5 s. Otherwise, it would be manually placed on the platform and allowed to stay for 20 s. Four spatial acquisition trials were performed in each day to let the rat entering the MWM from four different quadrants and the training session lasted for 5 consecutive days. On day 7, a probe trial was performed with the hidden platform removed. Rats were gently placed in the opposite quadrant to the original location of the platform and allowed to swim freely for 30 s. On day 6, the rats were allowed to have a rest. The latency to find the platform in spatial acquisition trials and the time spent and crossovers in platform quadrant in probe trials were recorded and analyzed using an animal behavior tracking system (EthoVision, Noldus Information Technologies, Netherlands). From days 31 to 35, another section of spatial acquisition trials was repeated followed with another probe trial on day 37. After finishing the behavior examination, the rats were sacrificed by over inhalation of isoflurane (ISO) with injection of potassium *via* the femoral vein. The timeline for MWM protocol is summarized in Figures 1B, 2A.

### Tissue preparation

On day 0 (before LPS or NS treatment, baseline), and days 3, 7, and 30 after LPS exposure, a cohort of animals (*n* = 8 for each time point each group) was selected for inflammatory cytokine examination. On the day of termination, rats were anesthetized by isoflurane (ISO, Baxter, Lessines, Belgium) prior to *vena cava* blood sampling (5 ml for each time). After 2 h of clotting at 4°C, blood samples were subjected to centrifugation at 1,000*g* for 20 min and the serum was later collected and stored at −80°C.

After decapitation, rat brain was quickly dissected and dividing by half using a chilled cold blade. The left part of the brain was rapidly grinded into small pieces and then homogenized by the RIPA Lysis Buffer (P0013C, Beyotime, Beijing, China) at the concentration of 20 mg tissue/200 μg buffer. Prior to homogenization, PMSF (100 mM, P0100-1, Solarbio, Beijing, China) was added to the RIPA lysis buffer to reach the concentration of 1 mM/ml. After centrifugation (Eppendorf, Centrifuge 5810R, China) at 1,000*g*, 4°C for 20 min, the supernatant was collected and stored at −80°C for further use.

### mRNA and RT-PCR

For the same rat, the right part of the brain was used for operating this procedure. Total RNA was extracted from hippocampus by Trizol reagent (Invitrogen, Paisley, United Kingdom) and was purified by the RNase Away Reagent (catalog number 18270466, Invitrogen, Paisley, United Kingdom). Prior the RT-PCR process, the integrity of mRNA was measured by NanoDrop 2000 (Thermo Scientific, Waltham, MA, United States) and the cDNA was synthesized by the M-MLV Reverse Transcriptase Kit according to the manufacturer’s instructions. The cDNA amplification was operated by the ABI 7500 Fast Real-Time PCR System (Applied Biosystems, Waltham, MA, United States) for 45 cycles (each for a duration of 2 min at 94°C, annealing for 5 s at 94°C and then for 30 s at 60°C and finally extension for 10 min at 72°C). The primer and amplification sequences for IL-1β, TNFα, and NF-κB were shown in Table 1. The level of mRNA expression was calculated by the SYBR Green direction method, and all the data were analyzed by the 2ΔΔCt method by the target gene expression in rat cortex corrected by β-actin expression.

**Table 1:** Primer sequences in RT-PCR.

mRNA	Forward primer (5′ → 3′)	Reverse primer (5′ → 3′)
IL-1β	5′-ATGAGAGCATCCAGCTTCAAATC-3′	5′-CACACTAGCAGGTCGTCATCATC-3′
TNFα	5′-CAAGAGCCCTTGCCCTAA-3′	5′-CAGAGCAATGACTCCAAAGTA-3′
NF-κB	5′-AATTTGGCTTCCTTTCTTGGCT-3′	5′-CTGCGATACCTTAATGACAGCG-3
β-actin	5′-CCCATCTATGAGGGTTACGC-3′	5′-TTTAATGTCACGCACGATTTC-3′

### Enzyme-linked immunosorbent assay measurements

On day 0 (before LPS or NS treatment), and days 3, 7, and 30 after LPS exposure, a cohort of animals (*n* = 8 for each time point each group) was selected for inflammatory cytokine examination (Figure 1). On the day of termination, rats were anesthetized by isoflurane (ISO, Baxter, Lessines, Belgium) prior to *vena cava* blood sampling. After 2 h of clotting at 4°C, the blood was centrifuged at 1,000*g* for 20 min and the serum was later collected and stored at −80°C. IL-1β and TNFα levels were measured using ELISA assay kits (RLB00 and RTA00, R&D Systems, Inc., Minneapolis, MN, United States) according to manufacturer instructions.

### Anesthesia on rats

The protocols for anesthesia have been published by Liu et al. elsewhere (Lu et al., 2012). Briefly, all rats were first inducted by ISO at the concentration of 3% followed by intramuscular injection of dexmedetomidine (DEX, Hengrui Medicine Co., Ltd., Jiangsu Province, China, 0.015 mg/kg). During the initial scanning, ISO (1%) in oxygen-enriched air was delivered *via* a customized nose cone with continuous intramuscular infusion of DEX (0.03 mg/kg/h). After the anatomical localization scans were acquired, the ISO concentration was decreased to 0.20%–0.25% with the respiration rate maintained at 60–85/min. When the respiration rate increased to 90/min, ISO concentration was adjusted to 0.5%. A small animal monitoring system (Model 1025, Small Animal Instruments Inc., New York, NY, United States), including a rectal temperature probe, respiration pneumonic sensor, and fiber optic oximetry sensor or cardiogram electrodes, was adopted for real-time monitoring. The core body temperature was maintained at 37°C *via* a warm water circulation system on the scanning bed.

### Magnetic resonance imaging

Animals to perform rs-fMRI (*n* = 10–12 for each group) scanning also performed MWM examination in order to minimize individual variations between rs-fMRI data and behavior performance. The rs-fMRI scanning was performed on days 3, 7, and 31 post-treatment and *prior to* the MWM test. (Figure 1B).

The animal MRI measurements were performed using the 7.0T Bruker PharmaScan System (70/16 PharmaScan, Bruker Biospin GmbH, Germany), operated *via* the ParaVision 5.1 software. The same coils, including a rat brain surface coil and a quadrature resonator volume coil, were adopted in all rats.

Anatomical images (T2WI) were acquired with fast-spin–echo sequence using TurboRARE with the following parameters: repetition time (TR) 5000.0 ms, echo time (TE) 36.0 ms, echo spacing 12 ms, echo-train length 8, field of view 3.50 × 3.50 cm, matrix size 256 × 256, and 28 slices with a thickness of 1.0 mm. For blood-oxygen-level-dependent (BOLD) images, EPI-SE-FOVsat sequence was used with the following parameters: matrix size 64 × 64, flip angle = 90°, resolution = 0.55 mm × 0.55 mm, 28 slices with a thickness of 1.0 mm, slice gap = 0, repetition time (TR) = 2000.0 ms, echo time (TE) = 18.0 ms, and volume = 180.

### Data processing and analysis

The data were pre-processed using spmratIHEP software (Nie et al., 2013; Liang et al., 2017) based on the statistical parametric mapping (SPM12; Welcome Department of Imaging Science);^1^ and Gretna software^2^ based on the Matlab (version 2014a, The MathWorks Inc., Natick, MA, United States). The data were carefully examined for completeness and truncation artifacts. All the functional images post-processing was performed by a single experienced observer, unaware to whom the scans belonged.

The voxel size of the functional datasets of all individuals was first multiplied by a factor of 5 to better approximate human dimensions, and then slice timing and realigning were performed. In the slice-timing section, the difference of slice acquisition times of each subject was corrected. Then, the realignment was done by adjusting the temporal-processed volume of each subject to the first volume in order to remove the head motion. After this, a mean image over the 180 volumes was recreated. In this section, data from subjects were filtered and preserved by which a 1 mm of translation in the *x*, *y*, or *z* axis and a 1° of rotation in each axis were set as criteria. Otherwise, it was eliminated. After spatial normalization into the Paxinos and Watson space, all the normalized images were re-sliced by 1.0 × 1.5 × 1.0 mm^3^ voxels (after zooming). Then the normalized functional series were smoothed with a Gaussian kernel of 2 mm^3^ Full Width at Half-maximum (FWHM) and the systematic drift or trend was removed using a linear model. Finally, the linear trended images were 0.01–0.08 Hz band-pass filtered and further corrected for the effect of head movement by regressing the translations and rotations of the head estimated during image realignment.

For default mode network (DMN) analysis, 12 regions of interest (ROIs; comprising left/right segmentation) were selected from the rat brain atlas image in Paxinos and Watson space (Nie et al., 2013; Liang et al., 2017). All these ROIs were summarized in Table 2.

**Table 2:** Region of interest in aged rat brain.

Region of interest (ROI)	ROI index	Short-form
01_Auditory cortex_ Left	1	L_AC
02_ Auditory cortex_ Right	2	R_AC
03_Hippocampus_Left	3	L_Hip
04_ Hippocampus_ Right	4	R_Hip
05_Orbital cortex_ Left	5	L_OC
06_ Orbital cortex_ Right	6	R_OC
07_Parietal association cortex_ Left	7	L_PAC
08_ Parietal association cortex_ Right	8	R_PAC
15_Prelimbic cortex_ Left	9	L_PrC
16_Prelimbic cortex_ Right	10	R_PrC
17_Retrosplenial cortex_ Left	11	L_RSC
18_Retrosplenial cortex_ Right	12	R_RSC

All the ROIs were drawn according to the Paxinos and Watsons space, with 12 ROIs (comprising left/right segmentation) were selected.

These ROIs were defined as nodes and functional connectivity (FC) was defined as the Pearson’s correlation coefficients (CCs) between each pair of nodes and calculated in Gretna. Weighted undirected 12 × 12 matrices were constructed for LPS and NS groups for each time point mentioned above, in which the strength of each connection was presented as FC.

### Graph theory analysis

Based on the weighted matrix, distance matrices of the above two groups were generated, where the distance between each node was defined as *L_ij_* = 1 − *w_ij_*. The *L_ij_* was identified as connection weight in nodes *i* and *j*.

Graph theoretical analysis using Gretna Toolbox^3^ was adopted to characterize FC patterns in group LPS and NS. For further analysis, global efficiency (*E*_glob_) and local efficiency (*E*_loc_) were used to assess network efficiency while nodal efficiency (*E*_i_) and nodal local efficiency (*E*_i_local_) were used to assess nodal centralities of the neural network. Based on graph theory, *E*_global_ is defined as the harmonic mean of the minimum path length between all possible pairs of nodes in neural network. *E*_loc_ is defined as the local efficiency of sub-graph composed of the neighbors of node *i*. *E*_i_ is defined as the ability of a node to propagate information with the other nodes in a network. *E*_i_local_ is defined as the efficiency of information propagation over a node’s direct neighbors (Wang et al., 2010). The mathematical definitions are characterized as the following:


$$\begin{array}{l} E_{\mathrm{glob}}\left(G\right)=\frac{1}{N\left(N-1\right)}\underset{i\neq j\in G}{\sum }\frac{1}{L_{i,j}},E_{loc}=\frac{1}{N}\underset{i\in G}{\sum }E_{\mathrm{glob}}\left(G_{i}\right),E_{i}\\ =\frac{1}{N-1}\underset{j\neq i\in G}{\sum }\frac{1}{L_{i,j}}\;\mathrm{and}\;E_{i\_\mathrm{local}}=\frac{1}{N_{G}\left(N_{G}-1\right)}\underset{j,k\in G}{\sum }\frac{1}{L_{jk}} \end{array}$$

where *L_i,j_* is the shortest path length between node *i*. *N* is the number of nodes in graph *G_i_*.

### Small-worldness analysis

The small-worldness property of neural network is described by the clustering coefficient (*C*_p_) and characteristic path length (*L*_p_). The *C*_p_ is defined as the average of clustering coefficients (*C*_i_) of all nodes in the neural network and the *L*_p_ is defined as the average of the shortest path length between any pair of nodes in the neural network. The *C*_p-rand_ and *L*_p-rand_ are defined as the mean *C*_p_ and *L*_p_ of the matched random network, respectively. The mathematical definitions are characterized as follows:


$$C\left(i\right)=\frac{2E_{\left(i\right)}}{K_{i}\left(K_{i}-1\right)}$$

where *E* is the number of existing connections among nodes *i* and *K* is the degree of nodes *i*. The threshold of statistical significance is presented by sparsity ranges of 0.1–0.5, where the step is 0.05. The network would be considered small-world if it meets the following conditions:


$$\gamma =C_{p}/C_{p-\mathrm{rand}}>1,\lambda =L_{p}/L_{p-\mathrm{rand}}\approx 1.$$

To identify differences in the FC matrix and topology properties between the two groups, two-sample *t*-test was performed. For multiple comparison correction, results in FC matrices were further corrected based on the network-based analysis (NBS) where the permutation tests were repeated 1,000 times. For results in topology properties, results were further corrected based on false discovery rate (FDR) correction. The value of *p* < 0.05 was considered statistically significant.

### Statistical analysis

Statistical analyses were conducted using IBM SPSS 25.0 statistics (IBM, Chicago, Illinois, United States). Values were presented as mean ± standard error of mean (SEM). Results in ELISA measurements and RT-PCR were analyzed by one-way ANOVA followed by *Bonferroni post hoc* analysis. Results in MWM probe trials were analyzed by two-sample *t*-test. Results in the MWM spatial acquisition trials were analyzed by repeated measures two-way ANOVA followed by *Bonferroni post hoc* analysis. The value of *p* < 0.05 was considered statistically significant. Analysis methods for rs-fMRI data are mentioned in the appropriate subsections above.

## Results

### Physiological factors during rs-fMRI scanning

Physiological factors during rs-fMRI data acquisition were strictly controlled. No differences were observed in weight, heart rate (HR), respiratory rate (RR) and blood oxygen level (SpO_2_) between the two groups (*p* = 0.838, *p* = 0.735, *p* = 0.676, *p* = 0.283, respectively, two-sample *t*-test; Table 3).

**Table 3:** Physiological factors during rs-fMRI data acquisition.

Days	Groups	Weight (g)	HR (/min)	RR (/min)	SpO_2_ (%)
3 days	NS	752.2 ± 25.3	295.5 ± 14.8	70.9 ± 2.0	99.0 ± 0.3
	LPS	744.9 ± 24.5	302.8 ± 15.4	72.3 ± 2.4	99.4 ± 0.2
7 days	NS	748.2 ± 25.1	307.5 ± 21.3	72.5 ± 2.6	99.5 ± 0.3
	LPS	702.4 ± 18.0	308.8 ± 19.0	73.6 ± 3.3	98.9 ± 0.4
31 days	NS	772.5 ± 25.8	307.3 ± 25.1	72.1 ± 2.2	99.4 ± 0.3
	LPS	740.1 ± 23.6	306.3 ± 17.0	72.9 ± 2.6	99.2 ± 0.3

Physiological factors were carefully controlled during data acquisition. Data were analyzed by two-sample *t*-test. The value of *p* < 0.05 was considered statistically significant.

### Lipopolysaccharide exposure caused short- and long-term impaired learning and spatial working memory

The MWM was used to determine the effects of LPS exposure on learning and spatial working memory in both short- (<7 days) and long-term (>30 days). From day 1 to day 5, significant differences were observed in rats exposed to LPS (*F*_(1,51)_ = 26.309 for treatment; *F*_(4, 204)_ = 73.179 for training days, *p* < 0.001, repeated measures two-way ANOVA; Figure 2B). A significant difference was also observed for interaction between training treatment and training days (*F*_(4, 204)_ = 2.840, *p* = 0.025). *Post hoc* analysis indicated that LPS caused significantly increased escape latency from day 2 to day 4 (*p* < 0.001, *p* = 0.003 and *p* < 0.001, respectively). For the probe trial on day 7, a shorter time spent in target quadrant and fewer platform crossovers (*p* < 0.001 and *p* < 0.001, respectively, two-sample *t*-test; Figures 2C,D) were observed in rats exposed to LPS. For the second acquisition trials from day 31 to day 35, significant differences were observed in rats exposed to LPS (*F*_(1,55)_ = 0.021 for treatment; *F*_(4, 220)_ = 6.109 for training days, *p* = 0.021 and *p* < 0.001, respectively, repeated measures two-way ANOVA; Figure 2E). A significant difference was also observed for interaction between training treatment and training days (*F*_(4, 204)_ = 2.704, *p* = 0.031). *Post hoc* analysis showed that rats exposed to LPS had longer escape latency on day 34 and 35 (*p* = 0.009 and *p* < 0.001, respectively). For the probe trial on day 37, a shorter time spent in the target quadrant and fewer platform crossovers were also observed for rats exposed to LPS (*p* < 0.001 and *p* < 0.001, respectively; Figures 2F,G).

### Lipopolysaccharide exposure caused increased systemic inflammatory response

**Figure 3:**
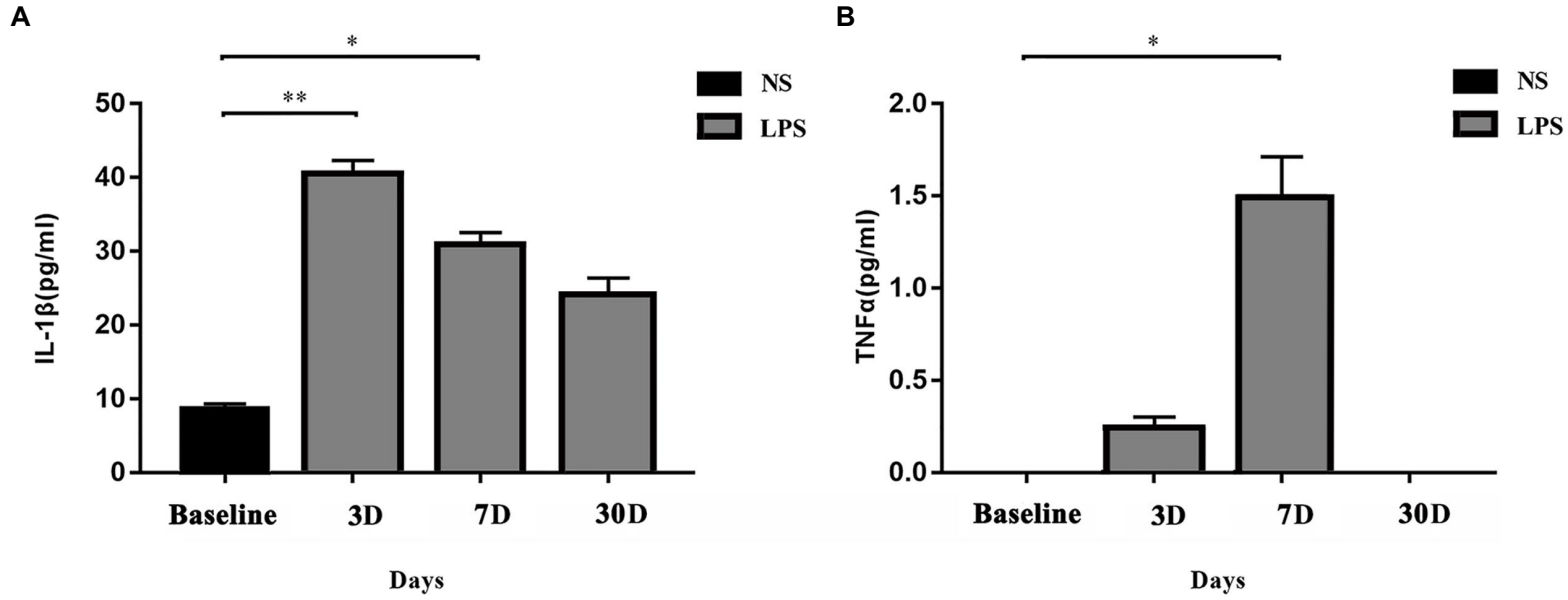
Systemic inflammatory responses. **(A)** IL-1β level was significantly elevated on day 3 and day 7 in rats exposed to lipopolysaccharide (LPS) as compared with rats treated with normal saline (NS). **(B)** TNFα level was significantly elevated on day 7 in rats exposed to LPS as compared with rats treated with normal saline NS. Data were presented as mean ± standard error of mean (SEM). ^*^p < 0.05, ^**^p < 0.01 vs. control group.

Systemic levels of IL-1β and TNFα were significantly increased in rats exposed to LPS (*p* = 0.002 and *p* = 0.009, respectively; one-way ANOVA). IL-1β level in rats exposed to LPS reached a peak at 3 days and remained elevated at 7 days (*p* = 0.001 and *p* = 0.04, respectively, *Bonferroni post hoc* analysis; Figure 3A). For TNFα level, a significant increase occurred at 7 days (*p* = 0.02, *Bonferroni post hoc* analysis; Figure 3B).

### Lipopolysaccharide exposure caused increased inflammatory response in rat brain

**Figure 4:**
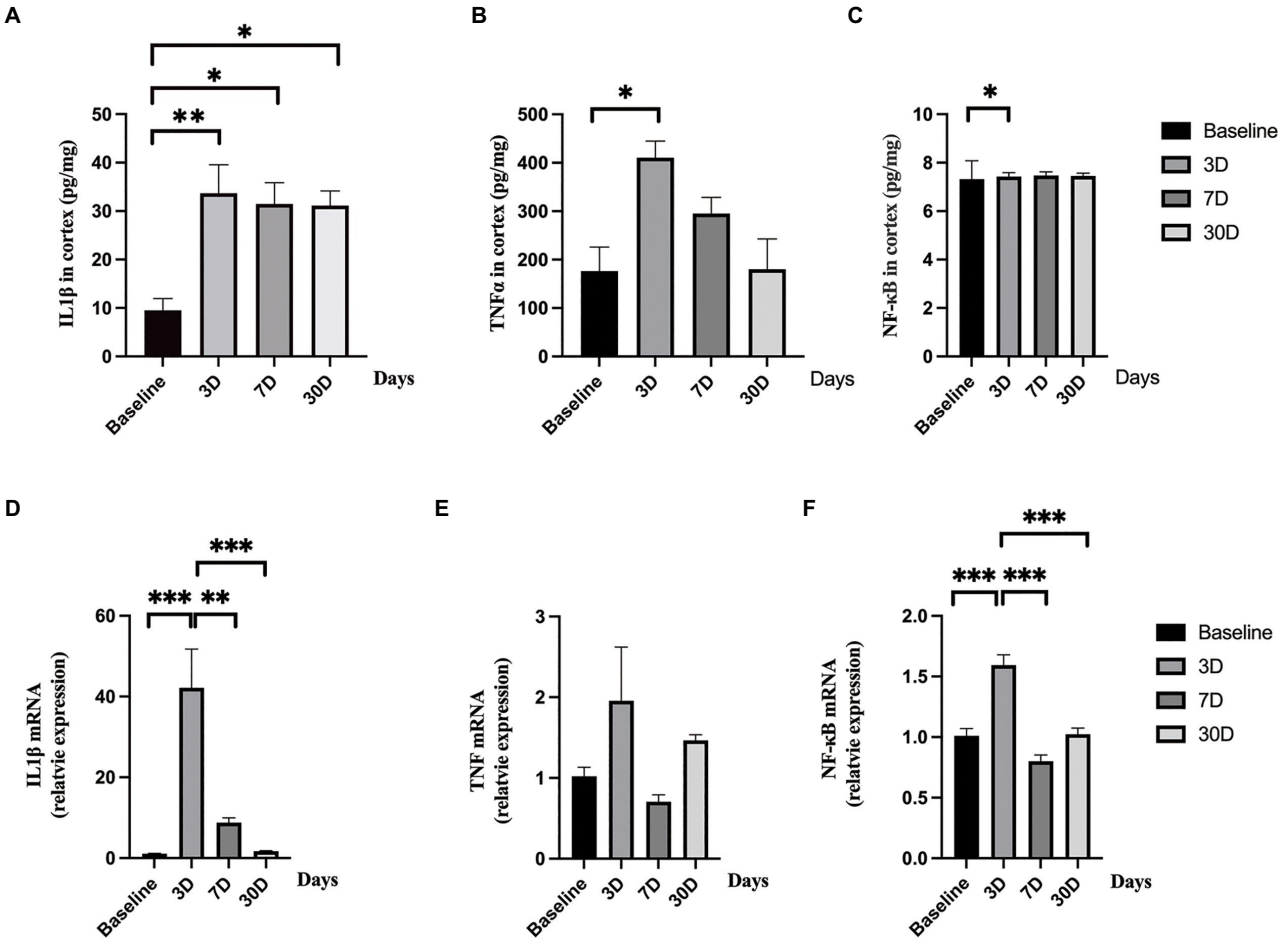
Central nerve system inflammatory responses. **(A–C)** The levels of IL-1β, TNFα, and NF-κB significantly elevated after lipopolysaccharide (LPS) exposure as compared with rats treated with normal saline (NS). **(D–F)** The mRNA levels of IL-1β and NF-κB significantly increased after lipopolysaccharide (LPS) exposure while the level of TNFα did not change as compared with rats treated with normal saline (NS). Data were presented as mean ± SEM. ^*^p < 0.05, ^**^p < 0.01, ^***^p < 0.001 vs. control group.

To determine whether LPS exposure caused inflammatory responses in aged rat brain, proinflammatory cytokines and mRNA expressions were measured. LPS exposure caused significant increase in IL-1β, TNFα, and NF-κB (*p* = 0.005, 0.005, and *p* = 0.043, respectively; one-way ANOVA; Figure 4). The secretion of IL-1β reached a peak level 3 days, slightly decreased on day 7, and remained significantly higher on day 30 after LPS exposure (*p* = 0.006, 0.023, and 0.026 as compared with control, *Bonferroni post hoc* analysis, Figure 4A). For TNFα and NF-κB, they both reached peak levels on day 3 after LPS exposure and went back to normal on day 7 and day 30 (*p* = 0.013, 0.477, and 1.000 as compared with control for TNFα; *p* = 0.043, 0.296 and 1.000 as compared with control for NF-κB, *Bonferroni post hoc* analysis, Figures 4B,C). When considering mRNA expression, LPS exposure caused a significant change in IL-1β and NF-κB (*p* < 0.001 and *p* < 0.001, Figures 4D,F) while no significant change was observed in TNFα level (*p* = 0.118, Figure 4E). The mRNA expressions of IL-1β and TNFα reached a peak level on day 3 after LPS exposure and fall back to almost baseline level on day 7 and day 30 (*p* < 0.001 for IL-1β and *p* < 0.001 for NF-κB, *Bonferroni post hoc* analysis).

### Lipopolysaccharide exposure caused significant short- and long-term FC alterations In aged rat DMN

**Figure 5:**
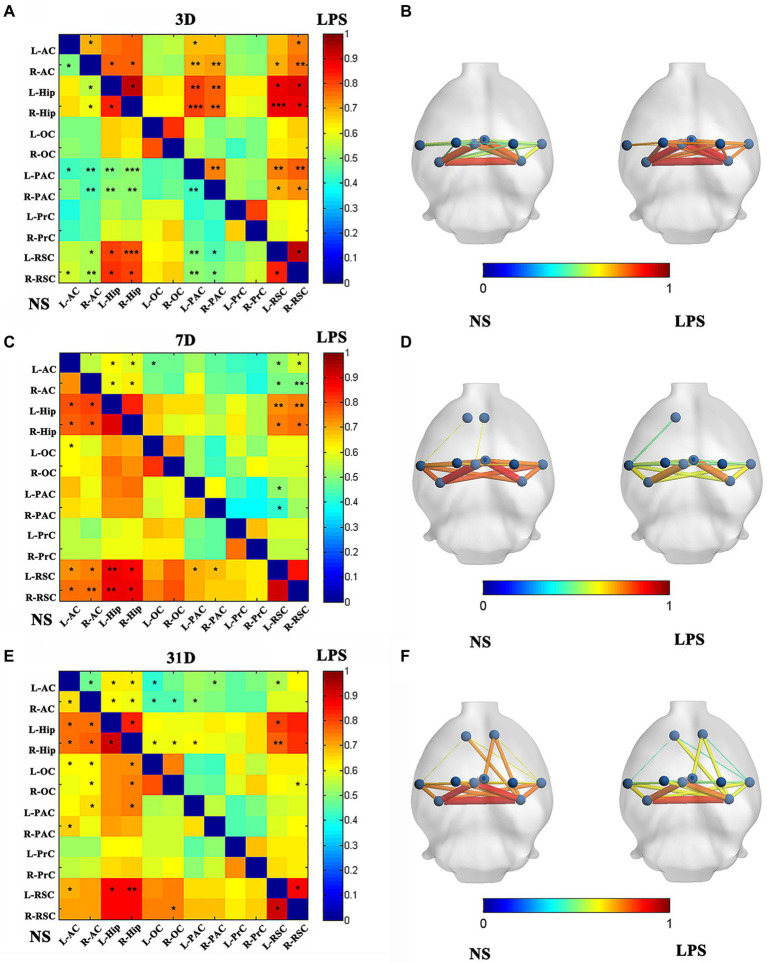
Functional connectivity (FC) in rats with lipopolysaccharide (LPS) exposure compared with control group. **(A,B)** FC value, nodes, and edges position in rats receiving LPS exposure (right) and control group (left) on day 3 after LPS exposure. **(C,D)** FC value, nodes, and edges position in rats receiving LPS exposure (right) and control group (left) on day 7 after treatment. **(E,F)** FC value, nodes, and edges position in rats receiving LPS exposure (right) and control group (left) on day 31 after treatment. Data were analyzed using two-sample *t*-test with network-based analysis correction (permutation 1,000 times). ^*^p < 0.05, ^**^p < 0.01, ^***^p < 0.001 vs. control group.

After LPS exposure, significant altered FC was temporarily observed with 13 in cortico-cortical connections and 10 in cortical–subcortical connections in LPS group on day 3 (two-sample *t*-test with NBS corrected; Figures 5A,B). One subcortico-subcortical connections were affected (L-Hip and R-Hip). On day 7, significant altered FC was observed in rats exposed to LPS with 7 in cortico-cortical connections and 8 in cortico-subcortical connections (two-sample *t*-test with NBS corrected; Figures 5C,D). On day 31, FC remained significantly altered in rats exposed to LPS with 9 in cortico-cortical connections and 9 in cortico-subcortical connections (two-sample *t*-test with NBS corrected; Figures 5E,F).

### Lipopolysaccharide exposure caused short-term instead of long-term topology alterations in aged rat neural network

**Figure 6:**
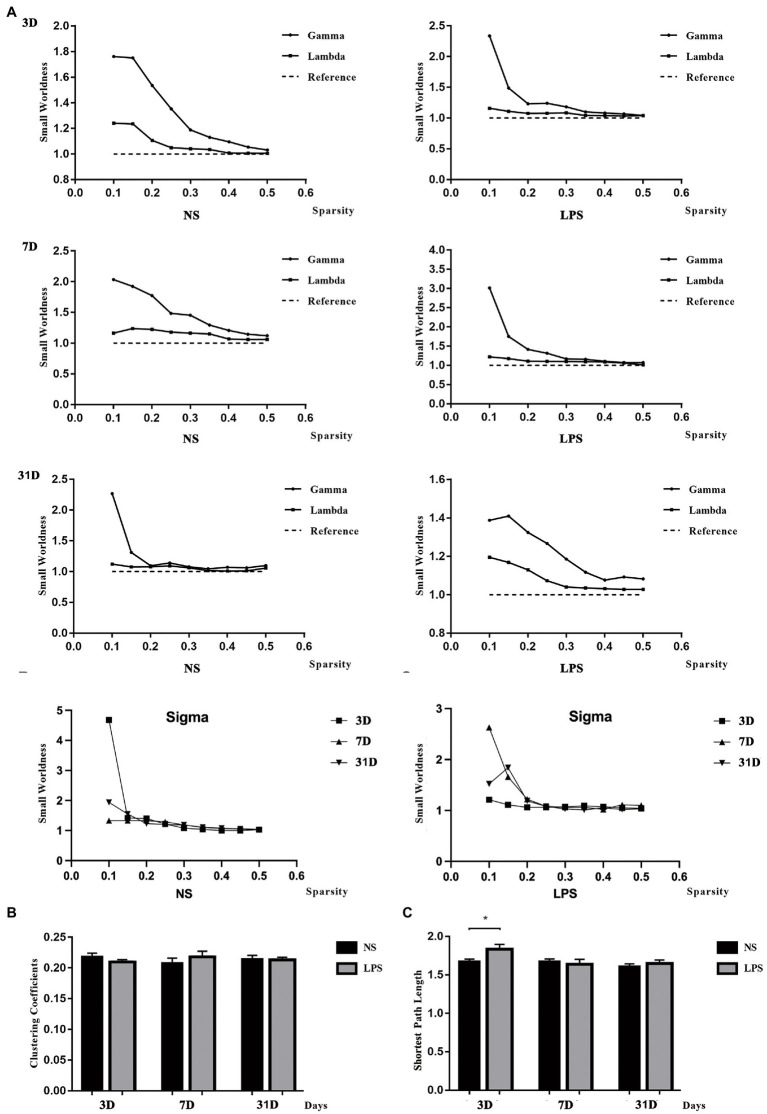
Small-worldness in aged rat default mode network (DMN). **(A)** The DMN in lipopolysaccharide (LPS) exposed and control rats showed the small-worldness at all time points. **(B)** LPS exposure caused no alteration in clustering coefficient (*C*_p_). **(C)** LPS exposure caused increased shortest path length (Lp) persisting for 3 days. Data were analyzed using two - sample *t*-test with false discovery rate (FDR) correction. ^**^p < 0.01 vs. control group.

All rats in both groups showed small-worldness properties for all time points (*γ* = *C*_p_/*C*_p-rand_ > 1, *λ* = *L*_p_/*L*_p-rand_ ≈ 1; Figure 6A). No significant differences were observed in clustering coefficient (Figure 6B) while a significant increased shortest path length was observed in rats exposed to LPS on day 3 (*p* = 0.00489, two-sample *t*-test with FDR corrected; Figure 6C).

**Figure 7:**
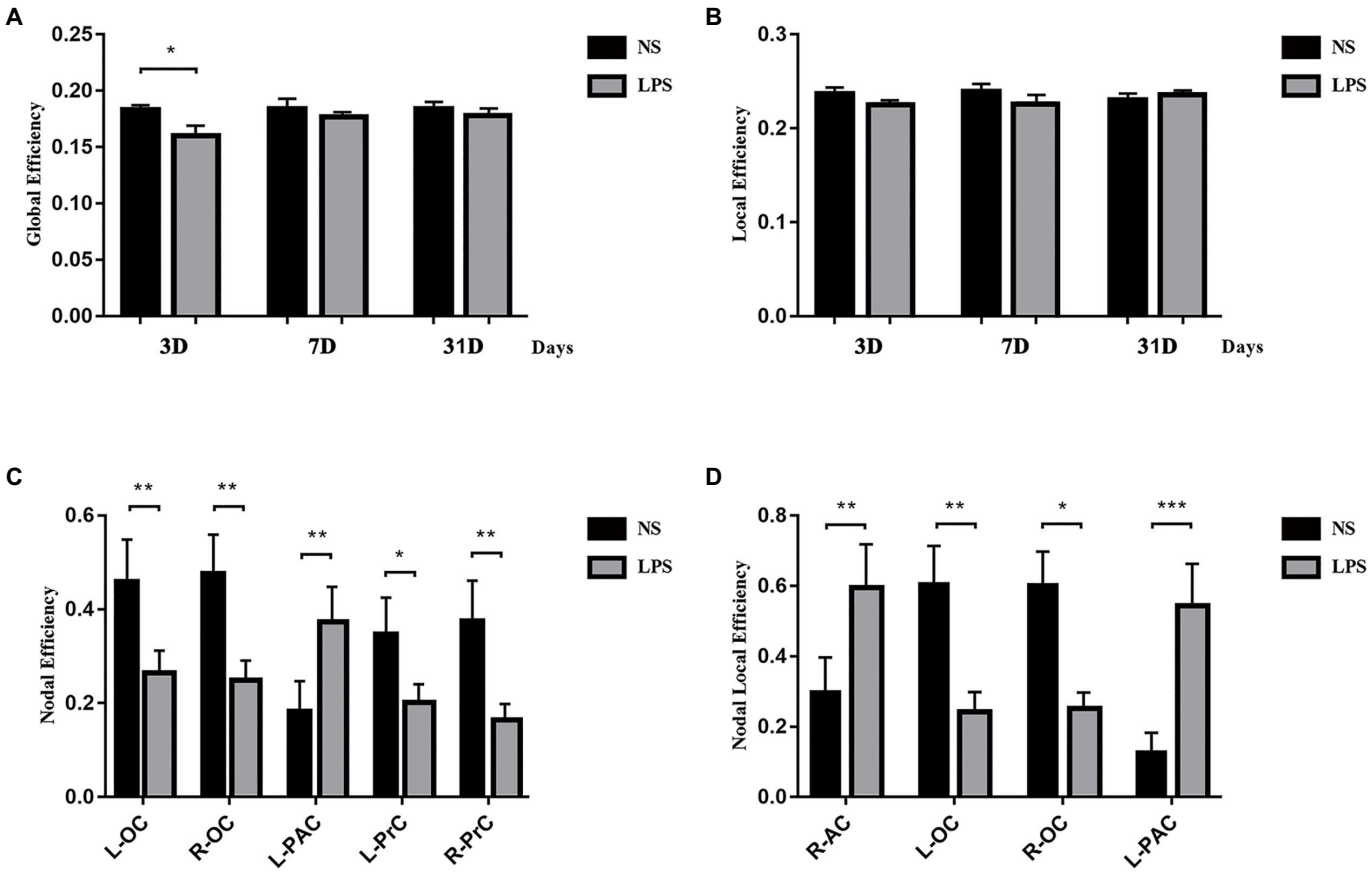
Topology structure alterations after lipopolysaccharide (LPS) exposure in aged rat brain. **(A)** Lower global efficiency was observed at 3 days in rats exposed to LPS. **(B)** No differences in local efficiency were observed. **(C)** Higher nodal efficiency in the left parietal association cortex and lower nodal efficiency in bilateral olfactory cortex and prelimbic cortices were observed in rats exposed to LPS. **(D)** Nodal local efficiency was observed higher in the right auditory cortex and left parietal association cortex and lower in bilateral olfactory cortex in rats exposed to LPS. Data were analyzed using two-sample *t*-test with false discovery rate (FDR) correction. ^*^p < 0.05, ^**^p < 0.01, ^***^p < 0.001 vs. control group.

Although lower global efficiency was observed at 3 days in rats exposed to LPS (*p* = 0.033, two-sample *t*-test with FDR corrected; Figure 7A), there was no difference between LPS-exposed and control rats in local efficiency (Figure 7B). For local properties, in rats exposed to LPS, *E*_i_ was significantly lower in the left and right occipital cortices (L-OC and R-OC) and left and right prelimbic cortices (L-PrC and R-PrC *p* = 0.0013, 0.0031, 0.019, and 0.0013, respectively, two-sample *t*-test with FDR corrected; Figure 5C). *E*_i_local_ was significantly lower in left and right occipital cortices (L-OC and R-OC; *p* = 0.0040 and 0.012, respectively, two-sample *t*-test with FDR corrected; Figure 5D). However, LPS exposure also caused higher *E*_i_ in left-parietal association cortex (L-PAC; *p* = 0.0054, Figure 7C), and higher *E*_i_local_ in right-auditory cortex (R-AC) and L-PAC (*p* = 0.0067 and 0.0001, Figure 7D). On days 7 and 31 after LPS exposure, no significant topology changes were observed between the two groups.

## Discussion

The present study demonstrated that inflammatory stress exposure in anesthesia caused altered intrinsic connectivity of the DMN, short- and long-term impairment in learning and spatial working memory, and altered short-term topology properties in aged rat brain.

Recent studies demonstrated the significance of neuroinflammation in postoperative cognitive decline (Subramaniyan and Terrando, 2019). Systemic inflammation induced by surgical trauma and neurotoxicity induced by anesthetic exposure have been proved to be major contributors (Liu et al., 2022). Our present study showed a significant increase in serum cytokines on day 3 (for IL-1β) and day 7 (for IL-1β and TNFα), demonstrating the occurrence of severe systemic inflammatory response. In central nerve system, significant increases of IL-1β (up to 30 days), TNFα (within 7 days), and NF-κB (within 7 days), indicating the existence of subsequent neuroinflammation. In order to validate the effect of neuroinflammation, the MWM was performed to test cognitive function in aged rats. The present study showed significant longer escape latency, lower time spent, and fewer crossovers in target quadrant in both MWM testing within 7 days and more than 30 days after LPS exposure, indicating that neuroinflammation induced short- and long-term cognitive dysfunction, which is consistent with our previous findings (Fu et al., 2014; Kan et al., 2016).

Altered FC in neural network is a major characteristic in cognitive dysfunction in both clinical practice (Labrenz et al., 2016; Tani et al., 2018) and preclinical experiments (Anckaerts et al., 2019; Passamonti et al., 2019). In the present study, we observed altered intrinsic connectivity in aged rat DMN on day 7 and day 31 after LPS exposure, which is consistent with clinical findings that FC in DMN is decreased in cognitive dysfunction (Qian et al., 2019; Zhao et al., 2020). Contrary to impaired MWM performance, increased intrinsic connectivity within the rat DMN was temporarily observed on day 3 after LPS exposure. Considering the severe systemic inflammation in aged rats, it could be explained by “systemic inflammation induced transient enhancement” as what was reported in human subjects 3.5 h after inflammatory exposure (Labrenz et al., 2016), which also shown that systemic inflammation began to affect DMN intrinsic connectivity at least 3 days after exposure.

Aside from whole DMN, the Hip and RSC are two most important regions (Raichle, 2015). While both contributing to learning and memory, the Hip plays a more significant role on recent delay while the RSC is more significant in remote delay and trace conditioning (assessed by fear conditioning) in AD related studies (Todd et al., 2019). Although altered RSC-related FC (which corresponds to the post-cingulate gyrus in mankind), which was observed in Aβ-induced cognitive decline in human subjects (Yamashita et al., 2019), was found in our study, the Hip rather than RSC seemed to play a more significant role in the long-term (11 vs. 12 CCs, 8 vs. 10CCs, 10 vs. 5 CCs for 3 days, 7 days and 31 days, respectively). We hypothesize that the reason for this may be a greater sensitivity of the Hip to inflammation in the aged rat as compared with normal adult rats (Barrientos et al., 2015). This result demonstrates that surgery stress and anesthetic neurotoxicity-induced cognitive decline differs from dementia induced by Aβ accumulation, supporting the recent idea that postoperative cognitive dysfunction (POCD) and AD are associated with different transcriptome changes despite their similar clinical manifestations (Wang et al., 2022).

Meanwhile, significant Hip-related FC changes combined with impaired behavior performances existed throughout the experiment period while not all the FCs in the DMN remained changed. This finding indicated that Hip played a significant role not only in neurodegeneration but also in inflammation exposure-induced cognitive dysfunction. Our findings are consistent with previous study that the hippocampus remained severe inflammatory response in long-term cognitive dysfunction (Li et al., 2020). Impaired Hip-related FCs demonstrated neural transmission dysfunction between Hip and other DMN regions, which indicated that impaired correlation between Hip and other DMN regions might be a significant character in inflammatory-induced cognitive dysfunction. Our previous study also showed that there are significant changes in Hip-related FCs in aged rat brain although treatment had already given (Liu et al., 2021). These findings all pointed to the significance of anti-inflammatory treatment in clinical practice, which may help to prevent long-term cognitive dysfunction after anesthesia and inflammation exposure.

Besides intrinsic connectivity (FC in the DMN), topology structure in neural network is also significant in information transmission. Small-worldness, a model combining high *C*_p_ and short *L*_p_, is a highly optional choice for studying complex networks in the setting of psychophysiological condition (Bassett and Bullmore, 2017; Miraglia et al., 2020). In the present study, rats with inflammatory exposure showed increased *L*_p_ followed with impaired behavior performances, which is similar with clinical findings that AD patients have disrupted small-worldness properties characterized by increased *L*_p_ (Dai et al., 2019; Lella and Estrada, 2020). However, it is different that the *C*_p_ is not changed in rats with inflammatory exposure-induced cognitive dysfunction as clinical studies reported that AD patients also have decreased *C*_p_ (Wu et al., 2019; Rauchmann et al., 2021). Interestingly, a lower *E*_global_ was observed 3 days after LPS exposure while no change in *E*_loc_ was also observed in the present study. These results indicated that inflammatory stress-induced cognitive dysfunction mainly relies on causing more steps in information exchange in small-worldness neural network instead of decreasing local interconnectivity between different regions, although they all disrupt small-worldness properties. This also supports the idea that POCD induced by anesthesia and surgery is different from early stage of AD. Whether the POCD develops into AD in the future still needs further studies. For regional properties (*E*_i_, *E*_i_local_), LPS exposure caused both increased and decreased changes, which may be a compensation for the decrease in global information transformation. On day 7 and 31 after LPS exposure, no significant differences could be observed in topology properties, while the difference in FC remained, showing that FC alterations, rather than topology changes, may last longer in LPS-induced cognitive impairment.

Based on the results of our study, we presented a background of DMN intrinsic connectivity and topology structures after inflammatory exposure in aged rat brain, indicating short- and long-term changes in functional connectivity and topology structures. Meanwhile, our study also pointed out brain regions that played a significant role in long-term cognitive dysfunction. In the future, a treatment could be given to these rats to evaluate the effect on aged rat DMN and cognitive function in both short- and long-term, providing further evidence for clinical practice. Considering that the DMN exists across species and the components are similar, these results can be used as background data, helping to judge treatment effect and drug-target design.

There are several limitations for the present study. First, the data were collected from aged rats instead of elderly patients. The reason for doing this is that using LPS on senior mankind for inducing sepsis and long-term cognitive dysfunction may cause ethical problem. Fortunately, recent studies reported that the component of DMN in aged rat is similar with those in human beings (Smallwood et al., 2021) and the FC alteration was also observed in various conditions (Serra et al., 2020), making the aged rats an optional choice for mechanism studying. Second, no data from post-mortem subjects were acquired for background comparison. The reason for not doing this is that we have to acquire longitudinal data and the rats have to undergo MWM testing. Third, the anesthesia method may have potential influences on FC patterns in the rat DMN (Paasonen et al., 2018). However, after comparing different methods, the combination of ISO and DEX was selected because it is a proven method for DMN studies and better preserves DMN connectivity as compared with several other anesthetic methods (Lu et al., 2012; Paasonen et al., 2018).

## Conclusion

The present study demonstrated inflammatory exposure in anesthesia management impairs intrinsic FC and disrupts small-worldness in aged rat DMN. Alterations in FC may be long-term while disrupted small-worldness may only be temporary. These results offer further insights into drug target design and new biomarkers for cognitive dysfunction induced by anesthesia and inflammatory stress exposure in clinical practice.

## Data availability statement

The raw data supporting the conclusions of this article will be made available by the authors, without undue reservation.

## Ethics statement

The animal study was reviewed and approved by Ethical Committee of Capital Medical University.

## Author contributions

YL, HF (Huiru Feng) and TW: study design. YL and HF (Huiru Feng): study performance. YL, HF (Huiqun Fu) and BN: rs-fMRI data analysis. YL and HF (Huiru Feng): manuscript writing. YW, BN and TW: manuscript revision. Dr. Yang Liu and Dr. Huiru Feng contributed equally to the manuscript and shares first authorship.

## Funding

This work was supported by grants from Beijing Municipal Health Commission (Jing 2019-2); Beijing municipal administration of hospitals clinical medicine development of special funding support (Code: ZYLX201706); and Beijing 215 high-level healthcare talent plan academic leader (Code: 008-0027).

## Conflict of interest

The authors declare that the research was conducted in the absence of any commercial or financial relationships that could be construed as a potential conflict of interest.

## Publisher’s note

All claims expressed in this article are solely those of the authors and do not necessarily represent those of their affiliated organizations, or those of the publisher, the editors and the reviewers. Any product that may be evaluated in this article, or claim that may be made by its manufacturer, is not guaranteed or endorsed by the publisher.

## Footnotes

^1^ http://www.fil.ion.ucl.ac.uk/spm

^2^ https://www.nitrc.org/projects/gretna/

^3^ https://www.nitrc.org/projects/gretna
